# Mathematics Self-Concept in New Zealand Elementary School Students: Evaluating Age-Related Decline

**DOI:** 10.3389/fpsyg.2019.02307

**Published:** 2019-10-16

**Authors:** Penelope W. St J. Watson, Christine M. Rubie-Davies, Kane Meissel

**Affiliations:** School of Learning, Development and Professional Practice, Faculty of Education and Social Work, The University of Auckland, Auckland, New Zealand

**Keywords:** mathematics self-concept, gender, ethnicity, age, career choice

## Abstract

The underrepresentation of females in mathematics-related fields may be explained by gender differences in mathematics self-concept (rather than ability) favoring males. Mathematics self-concept typically declines with student age, differs with student ethnicity, and is sensitive to teacher influence in early schooling. We investigated whether change in mathematics self-concept occurred within the context of a longitudinal intervention to raise and sustain teacher expectations of student achievement. This experimental study was conducted with a large sample of New Zealand primary school students and their teachers. Data were analyzed using longitudinal multilevel modeling with mathematics self-concept as the dependent variable and time (which represents students’ increasing age each year), gender, and ethnicity entered as predictors and achievement in mathematics included as a control variable. Interaction terms were also explored to investigate changes over time for different groups. All students demonstrated a small increase in mathematics self-concept over the 3-year period of the current study but mathematics self-concept was consistently greater for boys than girls. Māori, Asian, and Other students’ initial mathematics self-concept was higher than that of New Zealand European and Pacific Islanders’ (after controlling for achievement differences). However, a statistically significant decline in mathematics self-concept occurred for Māori students alone by the end of the study. The expected age-related reduction over time in student mathematics self-concept appeared to be mitigated in association with the longitudinal study. Nevertheless, the demonstration of a comparatively lower mathematics self-concept remained for girls overall and declined for Māori. Our results reinforce implications for future research into mathematics self-concept as a possible determinant of female student career choices.

## Introduction

Females remain underrepresented in science, technology, engineering, and mathematics (STEM) fields, which have been traditionally considered masculine domains (Watt, 2010). This *status quo* exists concurrently with a shortage of a skilled STEM labor force (Jacobs, 2005) and counter to evidence that suggests gender diversity in the workplace is beneficial (Woolley et al., 2010). Further, despite demonstrating that their mathematics ability is equal to that of males (OECD, 2015), women choose careers in fields outside STEM and a gendered wage gap, disadvantaging them, continues (Sansone, 2016). Gender differences in elementary school students’ mathematics confidence are larger than those for interest and achievement (Ganley and Lubienski, 2016). Moreover, although there are small gender differences in mathematics achievement, gender disparities in student career choice are large (Lindberg et al., 2010). Globally, a persistent gap in mathematics self-concept favoring males has been offered as a central explanation for differences in gendered STEM participation and, importantly, self-concept rather than ability appears to comprise the critical filter in career choice (Schoon, 2015). Mathematics self-concept has also differed by student ethnicity and has declined as student age increases (Wilkins, 2004). Socialization processes and interactions with significant others (e.g., teachers) have been posited as central to shaping students’ gendered expectations of success and the value they attribute to specific fields (Schoon, 2015). Further, a self-concept that embraces male mathematics superiority has originated from cultural beliefs and expectations (Correll, 2001). Notably, self-concept may be especially vulnerable to the influence of teachers in the early years of schooling (Petersen and Hyde, 2014). Teacher expectations have been influenced by beliefs (including socialized gender beliefs) about students (Dusek and Joseph, 1985). We explored, therefore, whether an intervention in which teachers were trained in high expectation practices might be associated with mitigating differences in student mathematics self-concept by gender and ethnicity, and might ameliorate its decline with increasing age.

The current research reports on data situated within the context of a 3-year intervention study conducted longitudinally with a large sample of New Zealand elementary school students (*n* = 1,739) from a range of schools (*n* = 11). This wider study aimed to raise and sustain teachers’ expectations of their students’ academic achievement (see Rubie-Davies et al., 2015 for a full account), and was conducted with the student participants and their teachers. The main study provided professional development to all teachers in the project. The pedagogical approaches associated with high expectation practices were targeted with the aim of supporting all participant teachers to develop these practices. These approaches included mixed ability grouping for learning activities (Rubie-Davies, 2008), fostering a warmer socioemotional climate in the classroom, promotion of positivity toward and between students, the development of mastery goals, enhancing collaborative classrooms, and facilitation of student autonomy (Rubie-Davies and Peterson, 2011).

The current study drew on the self-concept data collected as part of the wider professional development project. Specifically, we assessed whether student mathematics self-concept varied for students overall, and by student gender and ethnicity.

### Self-Concept

Byrne and Shavelson (1986) defined self-concept as one’s own perception of one’s abilities and efficacy. Specifically, self-efficacy (one’s belief about one’s ability to succeed in tasks) could be shaped by mastery and vicarious experiences, verbal persuasion, and one’s psychological and affective state (Bandura, 1997). Further, a sense of self-confidence, self-esteem, and acceptance of self have also been subsumed within self-concept (Marsh and Scalas, 2011) and have formed the basis for perceptions of possible selves (Franken, 1994). Self-concept is multi-dimensional and hierarchical in nature, develops early in a child’s educational career, and is positively associated with achievement (Chapman et al., 2000). Moreover, self-concept is influenced by experiences gained within one’s environment including social comparison and evaluations by significant others (Bong and Skaalvik, 2003), and is continually re-appraised and reinforced by intra-personal inferences (Bong and Clark, 1999). As a sense of identity and awareness of others develops, one’s self-concept (including domain-specific self-concept) tends to suffer a decline as a result of peer-comparison (Anderman and Maehr, 1994). Importantly, self-concept may be operationalized differently across cultures (Oettingen and Zosuls, 2006). Thus, examining self-concept within different contexts offers the opportunity to understand variations in student motivation and self-beliefs between cultures (Chiu and Klassen, 2008).

### Mathematics Self-Concept

Mathematics self-concept has been defined as one’s beliefs about one’s competence in mathematics (Ireson and Hallam, 2009). Further, mathematics self-concept has been positively associated with mathematics achievement (Ireson and Hallam, 2009), and student ratings of their skill, enjoyment of, and interest in mathematics (Erdogan and Şengul, 2014). One’s perceived ability to achieve well and one’s confidence in mathematics have also been associated with mathematics self-concept (Reyes, 1984). Moreover, it has been suggested that mathematics self-concept has been associated with individuals’ willingness to engage in quantitative scenarios (Eccles, 1987; Schoon, 2015).

#### Mathematics Self-Concept and Age

Mathematics self-concept has been shown to decline with schooling age (Wilkins, 2004) paralleling the age-related decline in general self-concept attributed to peer comparison (Anderman and Maehr, 1994). Further, the pattern of age-related decline has been supported in multiple contexts including that of New Zealand (Bonne, 2016).

In a study comprising a large number of students from the wider metropolitan area of Sydney, Australia (Grades 2–9, *n* = 3,679; Grades 7–11, *n* = 3,073, and 15 years of age and older, *n* = 1202), Marsh (1989) reported declines in mathematics self-concept in pre-adolescence and early adolescence (although finding some support for an increase in the construct’s levels during late adolescence). Further, of all the subject domains, the decline in academic self-concept was sharpest in mathematics and at the beginning of middle school (Marsh, 1989). Furthermore, Marsh (1989) stated that his study’s findings corroborated a consistent decline in mathematics self-concept with age found across numerous previous studies that had employed a range of robust instruments.

Fredricks and Eccles’ (2002) study with 514 students from an urban area in the Midwest of the US, ranging from Grades 1–12 (50% female; predominantly White American) further confirmed earlier findings (e.g., Marsh, 1989; Eccles et al., 1993) that perceptions of mathematics ability declined as grade level progressed. Fredricks and Eccles (2002) offered several explanations for the decline in belief in mathematics competence. In natural developmental patterns, for example, younger children’s views of their competence are somewhat more optimistic but as children grow older, comparison with peers incites a more realistic self-concept (Fredricks and Eccles, 2002). In addition, competitiveness increases and the nature of assessments changes through into adolescence (Fredricks and Eccles, 2002). Further, elementary school student evaluation is often more mastery-goal oriented, whereas at middle and secondary school, normative and socially comparative marking systems will more frequently be employed (Eccles et al., 1993).

Further research supported the decline of mathematics self-concept over time with US children. Jacobs et al. (2002) findings (resulting from a longitudinal study of 761 predominantly White American Grade 1–12 students in a large Mid-western city) showed a decline in mathematics self-competence perceptions over the course of schooling. A sharp decline in mathematics self-beliefs about mathematics competence beginning at middle school had been reported in some previous research conducted over shorter periods (e.g., Marsh, 1989). Jacobs et al. (2002), however, revealed a steady decline of mathematics self-beliefs over the 12 years of their longitudinal study. Marsh and Ayotte’s (2003) findings in their study of 1,103 French-speaking preadolescent Canadian children in Grades 2–6 further supported the pattern of the decline in math self-concept over time, within an additional context. Moreover, the findings of a longitudinal cohort-sequential study design conducted in Australia, Germany, and the US (Nagy et al., 2010) further endorsed the aforementioned pattern of decline. The authors found that for three cohorts of predominantly white Australian (*n* = 1,333), German (*n* = 4,688), and US (*n* = 2,378) secondary school students, mathematics self-concept declined across time. Erdogan and Şengul (2014) found the same pattern of decline in mathematics self-concept in a further context (Istanbul, Turkey) in a year-long study of 281 primary to secondary school students (Grades 4–6).

Relatively recent New Zealand data confirm a decline in mathematics self-beliefs (including mathematics self-concept) for New Zealand school-age children (Bonne, 2016). Bonne (2016) reported the findings of five New Zealand studies of student mathematics self-beliefs, comprising large numbers of children from early elementary to the end of middle school. From her synthesis of the findings, Bonne (2016) advocated for the power of teachers’ influence in improving mathematics self-beliefs.

The results of Wilkins’ (2004) large-scale study across multiple national contexts provided overarching evidence that mathematics self-concept declines with age. The author’s findings were based on data drawn from the TIMSS study comprising 290,000 students in early adolescence from 41 countries. Further, Wilkins (2004) shed light on the little-explored intersectionality of mathematics self-concept with other demographic characteristics, for example, gender. Boys, for example, have been found to hold higher levels of mathematics self-concept than girls, albeit that levels of the construct diminished as age progressed for both genders (Marsh, 1989; Wilkins, 2004).

#### Mathematics Self-Concept and Gender

In terms of differences in the rigidity of gender role between cultures, more masculine cultures (e.g., Austria, see Hofstede, 2003) have been associated with an avoidance of careers that are considered gender-role inappropriate (Chiu and Klassen, 2008). Such cultural attitudes could be suggested to limit the engagement of females, for example, in STEM fields. With specific regard to mathematics, girls have been shown to underestimate their ability and have typically expected less success in mathematics than boys (Eccles, 1987; Wang and Kenny, 2014). Notably, female New Zealand elementary and middle school students underestimated their ability even when their mathematics achievement was higher than that of their male peers (Bonne, 2016). In a study of German 8-9-year olds, Dickhäuser and Meyer (2006) found that girls attributed success in mathematics less to high ability and failure in the subject more to low ability than boys. Similarly, in a meta-analysis of cross-national gender differences in mathematics using Trends in International Mathematics and Science Study (TIMSS) and Program for International Student Assessment (PISA) data, Else-Quest et al. (2010) determined that boys showed more positive levels of mathematics confidence in almost all the participating countries.

In societies where gender-role rigidity was found to be greater (termed “masculine”) girls’ perception of themselves as mathematicians seemed to be further complicated (see Hofstede, 2003). Within such societies, girls were likely to value learning mathematics skills less, invest less effort and time in the subject, learn and achieve less mathematics, and have a lower mathematics self-concept than boys (Wigfield et al., 2004). Such scenarios have fostered girls’ self-exclusion from mathematics and have resulted in boys’ attainment of higher mathematics self-concepts despite their concurrent lower mathematics achievement (Chiu and Klassen, 2008).

The importance of differences in gender norms by cultural context for mathematics self-concept was underlined in further research. A gender gap in mathematics self-concept was found in Nagy et al. (2010) cross-national longitudinal study, and Erdogan and Şengul (2014) year-long study of Turkish students. Nagy et al. (2010) reported that boys’ level of mathematics self-concept was consistently greater than that of girls across the cultural contexts of Germany, Australia, and the US, although mathematics self-concept declined across time for both genders. The gender gap in mathematics self-concept was largest, however, for the German cohort when compared to the Australian and US cohorts (Nagy et al., 2010). This difference occurred in parallel to higher levels of gender-norm differentiation (reflective of the gender-role rigidity noted by Hofstede, 2003) found in Germany than in Australia and the US (see Williams and Best, 1990).

Importantly, mathematics self-concept is needed, in addition to content knowledge, to ensure persistence in trajectories leading to STEM fields (Ceci and Williams, 2009) and high levels of aptitude will not guarantee engagement in STEM without a mathematics self-concept that facilitates beliefs of success in the field (Goldman and Penner, 2014). Specifically, mathematics self-concept has been identified as a critical filter to female engagement in STEM fields and low levels of mathematics self-concept has been used to explain women’s underrepresentation in those fields (Schoon, 2015).

#### Mathematics Self-Concept and Ethnicity

Students in individualistic societies may place greater value on individuality engaging in downward comparison with peers and attaining a higher self-concept (Chiu and Klassen, 2008). Conversely, students in collectivist cultures seek upward comparison and consequently have a lower self-concept (termed as modesty bias; see White and Lehman, 2005). Where culture and ethnicity is concerned, however, interesting differences in mathematics self-concept have been found at national level (as opposed to student level) and between cultures.

Comparing national differences in the relationship between mathematics self-concept and achievement, Wilkins (2004) found that at the student level, higher mathematics self-concept was associated with higher mathematics achievement. At a national level, however, countries ranked with higher levels of achievement (e.g., Asian and East European countries) typically demonstrated lower mathematics self-concept. In contrast, countries ranked with comparatively lower achievement (e.g., Western Europe, the US, and Australia) registered higher mathematics self-concept. Students in individualistic societies (e.g., the US, Australia and New Zealand) may boost their mathematics self-concept by downward comparison with peers (Chiu and Klassen, 2008), perhaps explaining how higher self-concept was found in conjunction with lower achievement in mathematics. In contrast, students in collectivist cultures (e.g., East Asian cultures) have been socialized to value modesty and upwardly compare with peers in terms of mathematics self-concept, possibly explaining why, for them, a lower self-concept and higher achievement in mathematics are found simultaneously (White and Lehman, 2005). It could be suggested then that the culture of students may moderate the relationship between mathematics self-concept and performance.

In New Zealand, Caygill et al. (2013) found a positive association between attitude and achievement in mathematics, and mathematics self-confidence (linked to mathematics self-concept, see Reyes, 1984) was the strongest predictor of achievement for students within and between the nation’s major ethnicities (New Zealand European, Māori, Pacific Island, and Asian). Interventions to raise the mathematics achievement of New Zealand primary school students by improving their mathematics self-beliefs (see Bonne, 2016) have borne positive results. Research has been called for, however, that engages in conducting such explorations longitudinally (Bonne, 2016).

### Factors That Influence Mathematics Self-Concept

#### Socialization of Stereotypical Gender Beliefs

Socialization comprises interactions with the environment and significant others, including teachers and peers (Marsh, 1990a). Further, an awareness of cultural norms develops (Schoon, 2015) as children form an understanding of roles and behaviors that are appropriate for them through social learning (see Bandura, 2002). Specifically, in the New Zealand context, women have been associated in television advertisements with home-making and glamorous appearance, whereas men have been portrayed as physically and mentally tough, and technically agentic (Michelle, 2012). Such evidence provides support for the suggestion that New Zealand is a gender-essentialist society where expectations of conformity to gender roles is a powerful shaper of choices for young people (Cushman, 2008). Stereotypes can also reinforce relationships between ethnicity and gender. Māori women have been historically stereotyped as unsuitable candidates for mathematics-related fields in post-colonial New Zealand (McKinley, 2008). Further, although New Zealand European women have media dominance among New Zealand ethnic-racial groups, they are most frequently portrayed in roles that negate their agency in scientific and technical fields (Michelle, 2012).

Awareness of stereotypes and popularly held beliefs specific to perceptions of mathematics ability emerge in children between the ages of 7 and 12 years (Steele, 2003). Moreover, elementary school children are particularly receptive to stereotypical information (Gunderson et al., 2012). Thus, primary and middle school children’s concept of their mathematical selves has been potentially influenced by a stereotype in which mathematics has been historically associated with masculinity and purports the mathematical superiority of males (Li, 1999; Neugebauer et al., 2011).

Importantly, when stereotypes are activated, they have been known to be central in influencing how information is processed and subsequently affect behavior (Heyder and Kessels, 2016) confirming the underpinning stereotype (Hamilton et al., 1990). Further, gender stereotypes strengthen with age (see Martinot and Désert, 2007). One could suggest, therefore, that implications are presented for the future selves of women and particularly women from ethnic-racial minorities where stereotypes purporting the suitability of males and dominant ethnic-racial groups for STEM fields prevail.

#### Social Comparison

As mentioned above, social comparison (e.g., comparing performance or attainment with that of peers) influences self-concept (O’Mara et al., 2006). Where students are grouped according to ability, social comparison is significant in shaping students’ conceptualization of their own ability because of the opportunity to upwardly or downwardly compare (Burleson et al., 2005). Although New Zealand elementary classes are most frequently non-streamed (untracked), within-class ability grouping is endemic (Rubie-Davies, 2015). New Zealand elementary students have the opportunity then, to compare their ability upward and downward with that of their classroom peers. Self-concept can be boosted as a result of downward comparison but reduced by upward comparison (Burleson et al., 2005). Specifically, comparison with peers who have superior mathematics achievement is likely to promote a relatively negative mathematics self-concept (Bong, 1998).

#### The Specific Impact of Teachers

Teachers play an important role in shaping student self-concept. Teachers’ expectations of their students’ success (explained in more detail below), for example, is a central influence on student self-perceptions of ability and competence (Harris and Rosenthal, 1985). Specifically, the ability judgments of significant others (e.g., teachers) have held especial potential to impact mathematics self-concept (Dickhäuser and Stiensmeier-Pelster, 2003) and notably so for elementary school children (Marsh et al., 1998). Further, girls have relied on a perception of teacher evaluations of their mathematics ability to a greater extent than boys in forming attributions of their mathematics ability (Dickhäuser and Meyer, 2006). Erdogan and Şengul (2014) stressed the roles of quality of instruction and the classroom climate as central in influencing the development of students’ mathematics self-concept.

Teachers’ stereotypical beliefs about mathematics can influence students’ perceptions of their own mathematical ability (Tiedemann, 2000). Moreover, teacher expectations have mediated the relationship between such beliefs and student outcomes (Younger and Warrington, 2008). Nevertheless, although negative beliefs (e.g., about the gender-appropriateness of certain subject domains) can result in a non-adaptive interplay between beliefs and performance (Sansone, 2016), self-perceptions are malleable (Harackiewicz et al., 2012). Further, in as much as teachers have conveyed gendered beliefs about mathematics (Gunderson et al., 2012), they also have the power to strengthen students’ self-beliefs (Siegle and McCoach, 2007; Bonne, 2016). Importantly, Sansone (2016) suggested that a detrimental self-fulfilling prophecy caused by gender beliefs about mathematics could be disrupted by an improvement in teacher-student relationships and an enhanced climate of safety at school.

### Teacher Expectations of Students’ Academic Outcomes

Differential teacher expectations have been identified as one of several factors responsible for differences in students’ academic outcomes. Specifically, teacher expectations have resulted in their differential treatment of students (Brophy and Good, 1970), and consequent differences in student outcomes including the shaping of self-concept and motivation (Harris and Rosenthal, 1985). Page and Rosenthal (1990) suggested that teacher beliefs about student gender and race could influence teachers’ beliefs about student potential in quantitative endeavors. The expression of teaching behaviors associated with increased learning opportunities could thus result from teachers’ beliefs of certain groups’ superior aptitude and efficacy in mathematics (i.e., Asian and male students, see Page and Rosenthal, 1990). Student beliefs about their own STEM-field ability and competence, a heightened susceptibility to biased teacher expectations, and ultimately student academic outcomes differentiated by gender could, therefore, be results of endorsing the mathematics stereotype (Eccles and Wigfield, 1985).

It could be suggested then that teacher expectations based on stereotypical gender beliefs (founded on inaccuracy) have the potential to become self-fulfilling prophecies for students and promote inequity. Importantly, in New Zealand, teachers’ expectations of student academic success have been shown to be influenced by student ethnicity with less success expected for Māori students (Rubie-Davies et al., 2006). Messages conveyed by teachers via such expectations have been known to trigger stereotype threat (see Steele and Aronson, 1995), one outcome of which is dis-identification with specific domains (Woodcock et al., 2012).

Teacher expectations have been identified at both individual and class level, but were found to be most influential when applied to the whole class (Rubie-Davies, 2007). Further, clear distinctions can be drawn between teachers who have high or low expectations for *all* their students (Weinstein, 2002; Rubie-Davies and Peterson, 2011). Rubie-Davies and Peterson (2011) found that certain characteristics were associated with teachers who had high expectations for all the students in their class (e.g., use of mixed ability grouping, choice in learning experiences, positive social climate in the classroom, intrinsic motivation, and well-defined goals). In contrast, the characteristics associated with teachers who had low expectations for all students in their classes (e.g., use of ability groups, teacher-determined learning experiences, negative social climate in the classroom, extrinsic motivation, and uncertainty of learning direction) differed starkly from those of their high expectation colleagues.

### The Current Study

A need exists for longitudinal research regarding mathematics self-beliefs and more so given the ability of teachers to alter students’ self-beliefs via changes in pedagogy (Bonne, 2016). Whether changes in mathematics self-concept occurred over time was explored in the current study. The aforementioned research was conducted with New Zealand elementary school students during a 3-year longitudinal study evaluating an intervention to raise and sustain teacher expectations of student achievement in mathematics. Positive benefits of the intervention were reported for student academic outcomes (Rubie-Davies and Rosenthal, 2016) and success in modeling the practices of high expectation teachers (McDonald et al., 2014; Rubie-Davies et al., 2015). Further, the consequences of inaccurate teacher expectations for gifted readers (Garrett et al., 2015), the relationship between student ethnicity and teacher expectations (Rubie-Davies et al., 2013), and the influence of teacher gender on teacher expectations in mathematics and reading (Watson et al., 2016, 2017) have already been explored. The current study marked a starting point in exploring whether the intervention was related to changes in student self-beliefs. Specifically, student mathematics self-concept data were analyzed via multilevel modeling.

Previous research had found that teacher expectations could influence student self-beliefs (Harris and Rosenthal, 1985; Younger and Warrington, 2008) and that self-perceptions and expectations of success were malleable (Harackiewicz et al., 2012). On the basis of such research, we hypothesized that within the context of a 3-year longitudinal intervention focused on the development of beliefs and practices associated with teachers’ high expectations for all their students, an expected decline in student mathematics self-concept would be ameliorated (H1), a gender gap in mathematics self-concept favoring boys would be addressed (H2), and equitable mathematics self-concepts between students in different ethnic groups would be promoted (H3).

## Materials and Methods

The current study draws on data collected across 3 years of a longitudinal study that investigated teacher expectations of elementary school students to determine whether expectations could be raised and sustained and whether or not there were effects on student achievement outcomes (see Rubie-Davies et al., 2015). Socio-demographic factors (i.e., age, gender, and ethnicity) associated with different student self-reported levels of self-concept for mathematics were explored over time (to show any changes associated with an increase in age).

### Participants

Following ethical approval from the authors’ institutional ethics committee, 11 New Zealand elementary schools comprising a range of socioeconomic (SES) levels were recruited for the study. In New Zealand, school-based SES is based on a 10-point scale with 1 assigned to the poorest schools and 10 to the most affluent. Informed and written consent was gathered from parents/legal guardians in order to invite students to participate in the research. The current study comprised student participants (*n*_baseline_ = 1,739) drawn from the above schools. As the focus of the study was to report student outcomes, student, rather than school-based participant demographics, are presented (see Table 1). The proportions of male and female students are consistent with those found in the New Zealand scholastic population. The range and proportion of ethnicities comprising the current participant sample were similar to that represented in the school-aged population of the large urban area in which the study was conducted (see Table 1).

**TABLE 1:** Participant demographic information (student *n* at baseline = 1,739; school *n* = 11).

**Gender**	**Male**	**Female**				
	49.8%	50.2%				

**Ethnicity**	**NZ European**	**Māori**	**Pacific Island**	**Asian**	**Other**	

	47.4%	17.8%	16.2%	15.9%	2.7%	

**Age**	**7 years**	**8 years**	**9 years**	**10 years**	**11 years**	**12 years**

	8.1%	18.3%	19.2%	19.3%	17.5%	17.5%

### Measures

In addition to the sociodemographic factors explored, two main measures (described below) were used in this study, one to measure student self-concept in mathematics and one to measure student achievement. Descriptive statistics for the achievement and self-concept measures are shown in Table 2. Achievement and self-concept were both measured at the beginning and end of each of the 3 years. To account for the differing gaps between each time point, this was recoded into the number of school terms after baseline, with baseline entered as 0. There are four terms per academic year in New Zealand.

**TABLE 2:** Descriptive statistics for mathematics self concept and mathematics achievement.

	**Time**	***n***	**Mean**	**Std. Deviation**
Maths self concept	BOY 1^1^	1739	3.66	1.03
	EOY 1	1015	3.75	0.97
	BOY 2	672	3.68	0.96
	EOY 2	526	3.64	0.98
	BOY 3	266	3.88	0.93
	EOY 3	242	3.79	0.86
	Total	4460	3.70	0.98
Maths achievement	BOY 1	1739	–0.064	0.786
	EOY 1	1015	0.101	0.867
	BOY 2	672	–0.045	0.755
	EOY 2	526	–0.057	0.764
	BOY 3	266	–0.231	0.701
	EOY 3	242	–0.040	0.659
	Total	4460	–0.032	0.791

*^1^BOY, beginning of year; EOY, end of year.*

#### Self-Concept for Mathematics

Mathematics self-concept was measured using the mathematics self-concept subscale of the Self-Description Questionnaire-1 (SDQ-1; see Marsh, 1990b). Students responded to items (e.g., “Work in mathematics is easy for me”) on a Likert scale ranging from 1 to 5, where 1 = false; 2 = mostly false; 3 = sometimes false; sometimes true; 4 = mainly true; 5 = true. This scale was standardized before entry into the multilevel models in order to provide standardized coefficients. The alpha coefficient for the subscale (α = 0.93) gave confidence in using data generated by it in further analyses.

#### Student Achievement

Student achievement was measured using a standardized tool, e-asTTle mathematics (e-asTTle Project Team, 2009). National norming trials were conducted in 2009–2010 in order to establish the robustness of e-asTTle. All items in the e-asTTle system were calibrated using item response theory (IRT) scoring procedures (Embretson and Reise, 2000) and the standard error of measurement for any e-asTTle test was estimated to be 15 points, with a standard deviation of 100 for each year level (see e-asTTle website)^1^. The test-retest reliability of e-asTTle is reported to be α = 0.96 (Ministry of Education, and NZCER, 2012). Thus, confidence in test consistency was assured. Confidence was also assured in comparing student scores in relation to the normative expectation for their academic year level, regardless of the test level administered to students.

Because expected scores differ by year level, the published normative expectation for each year level was subtracted from each student’s overall total mathematics score, generating a “centered” score that indicated the distance from the norm, while retaining the metric of measurement. The average achievement in the current sample was approximately equivalent to that of the normative sample at baseline (*M* = −6.47, *SD* = 78.65, *N* = 1739).

This centering process shifts the mean but does not affect the standard deviation. Therefore, the sample variability was somewhat less than for the e-asTTle normative sample. To place e-asTTle on a comparable scale to the other variables, the scores were divided by the standard deviation for the tool, as recommended by Schagen and Hodgen (2009).

### Procedures and Missing Data

Data collection for this study occurred at the beginning and end of each academic year over a 3-year period commencing in February 2011. At each data collection point, student mathematics achievement and self-concept were measured using e-asTTle mathematics and the mathematics self-concept subscale of the SDQ-1, as described above. Paper and pencil versions of the questionnaire containing the SDQ-1 were administered by a researcher and research assistant to each class during the school day in their own classroom. Worksheets were provided for any student who opted not to participate, but were not needed in all but three cases. Grade–normed tests had been designed at each curriculum level within e-asTTle mathematics (e.g., Level 2, Level 2/3, Level 3, Level 3/4, Level 4, and Level 4/5), and were chosen by each teacher as was appropriate for the number of students per approximate levels of achievement of their class.

However, not all students provided data at each time point. Sometimes this was due to illness on the day of the data collection, but there were also structural issues that precluded data collection across the full 3-year period in some cases. For example, students in New Zealand often change schools at the end of Year 6 (∼11 years old) and most change again in Year 8 (∼13 years old). Although HLM procedures are able to incorporate complete case information, whereby a single missing time point is not a major concern, significant attrition over time can still be problematic. To reduce the possibility that our results would simply reflect students dropping out of the sample, we only included students for whom baseline results were available, and who were present for at least a complete year. We also evaluated the student achievement scores of the students included in the final sample, against those who were excluded, and found no statistically significant differences by student achievement (*M*_diff_ = 0.05 SD, *p* = 0.087). Analyses were carried out using IBM (2016) SPSS v 24.0 and MLwiN v 3.02 (Charlton et al., 2017).

### Analytical Procedures

The primary aim of the current study was to investigate whether, within the context of an intervention to raise and sustain teacher expectations of student achievement, an expected decline in student mathematics self-concept with age and gaps in the construct’s levels by gender and ethnicity would be observed. To examine this aim, three-level hierarchical linear models (HLM) were built. These models explored the association over time between sociodemographic factors (age, gender, and ethnicity) and standardized self-concept in mathematics (the dependent variable), controlling for measured student achievement in mathematics. HLM is a multilevel regression framework that allows dependency in the data to be considered. Students within a particular classroom, or attending a particular school, tend to have a degree of shared variance, meaning that educational data generally violate the independence assumption (Osborne, 2000). Within the HLM framework, students in the same school can be specified as being nested within schools, for example, with students at level one and schools at level two. As the current study included a time component, observations were treated as level one nested within students at level two, and schools treated as level three. An alternative specification could have included teachers as level three, but when an unconditional model was specified including the teacher level, the variance at this level was negligible (1.4%). Given the extra complexity of adding this level (particularly because students change teachers each year), the teacher level was not included in the final models.

As mathematics achievement had already been “centered” against the normative expectations, achievement was not centered further when added to the HLM. As noted above, these scores were standardized by dividing by the standard deviation for the tool. Ethnicity was entered as a polytomous categorical variable, with Māori, Pasifika (of Pacific Island origin), and Asian students treated as binary dummy variables, and New Zealand European students treated as the reference category because this was the largest group. Student gender was included as a dummy variable with boys treated as the reference category—in this case because we wanted to focus on the relations for female students. Interaction terms (see Aguinis et al., 2013) were also included to explore the change in self-concept over time for each group.

To control for individual differences in achievement at each time point, mathematics achievement was added to the model along with the variable for time. This procedure was carried out after fitting the three-level unconditional random intercepts model with mathematics self-concept as the dependent variable (see Table 3 for the final model). Age was also included in this model because prior research has indicated that self-concept tends to decline with age. This was followed by the variables of gender and student ethnicity as well as the interactions with time. Random slopes were tested for the effect of student achievement on self-concept but this did not improve the model fit so only random intercepts were used in the final model.

**TABLE 3:** Multilevel model exploring socio-demographic predictors of standardized mathematics self-concept within a New Zealand elementary student sample.

	**Unconditional model**			**Final model**		
**Parameter**	**β**	***SE***	***p***	**β**	***SE***	***p***
**Fixed effects**						
Intercept	0.007	0.069	<0.001	–0.101	0.067	0.132
**Time**				0.029	0.007	<0.001
e-asTTle Maths vs. Norm^†^				0.119	0.015	<0.001
Age (grand-mean centered)				–0.048	0.015	0.001
**Gender (ref. = Male)**						
Female				–0.197	0.043	<0.001
**Ethnicity (ref. = European)**						
Māori				0.209	0.062	<0.001
Pasifika				0.354	0.067	<0.001
Asian				0.448	0.064	<0.001
Other ethnicity				0.567	0.136	<0.001
**Interaction terms**						
Female student × time				–0.012	0.007	0.087
Māori student × time				–0.023	0.01	0.02
Pasifika student × time				–0.012	0.01	0.23
Asian student × time				–0.016	0.011	0.146
Other ethnicity × time				–0.038	0.023	0.098
Between school variance $(\sigma_{\text{v}\,0}^{2}$)	0.056 [6.07%]	0.025	0.034	0.034 [3.7%]	0.016	0.033
Between student variance ($\sigma_{\text{u}\,0}^{2}$)	0.542 [58.7%]	0.025	<0.001	0.461 [49.9%]	0.023	<0.001
Repeated measures variance ($\sigma_{\text{e}}^{2}$)	0.423 [45.8%]	0.012	<0.001	0.428 [46.3%]	0.012	<0.001
−2^∗^log likelihood	11,301.33			11,115.92		

*^†^e-asTTle scores were divided by 100, so parameter estimates reflect expected difference per 100 e-asTTle points. VPC shown in square brackets.*

## Results

On average, self-concept was relatively high across the 3-year period (*M* = 3.7, see Table 2). The results of the unconditional and final multilevel models are shown in Table 3 below. The change in deviance was 185.41 indicating that the final model was a statistically significantly better fit for the data than the null model. Using the method described by Bryk and Raudenbush (1992) to approximate the proportion of variance explained by the final model, we found that the model explained approximately 39.3% of the school-level variance, and 14.9% of the student-level variance. There was no reduction in level-one variance.

The results indicated that higher mathematics achievement was statistically significantly associated with higher self-concept for mathematics (β = 0.119, *p* < 0.001). Several other covariates were associated with self-concept, even after controlling for achievement differences (see Table 3).

Mathematics self-concept was negatively, and statistically significantly associated with age at each time point, with older students reporting significantly lower self-concept than younger students (−0.048 per year of age, *p* = 0.001; see Table 3). Surprisingly, however, there was a statistically significant increase over time within the current study (0.029 per time point, *p* < *0.001*), for the same students, despite a 3-year increase in age. Female students had statistically significantly lower self-concept for mathematics on average (−0.197, *p* < 0.001), and this gap remained throughout the duration of the study. Controlling for achievement, self-concept for mathematics was lowest among New Zealand European students, with all other ethnicities having statistically significantly higher self-concept at baseline. However, the reported self-concept for New Zealand European students (the reference group for ethnicity) increased more over time than that for all other ethnicities (see the interaction terms in Table 3); though the relative decline was statistically significant only among Māori students during the 3-year period (−0.023 per time point, *p* = 0.02).

## Discussion

We expected that we would see the typical decline in student mathematics self-concept ameliorated within the context of an intervention focused on the development of beliefs and practices associated with teachers’ high expectations for all their students (H1). We also hypothesized that the intervention would address a gender gap in mathematics self-concept favoring boys (H2) and would promote equitable mathematics self-concepts in students across different ethnic groups (H3). The well-evidenced age-related decline in mathematics self-concept appeared to be diminished over the course of the 3-year period, supporting (with reservations that are described below) the first hypothesis. Equitable levels of mathematics self-concept were not achieved, however, for gender or across ethnic groups resulting in a lack of support for the second and third hypotheses.

Although older students’ mathematics self-concepts were lower at baseline than younger students’, with statistical significance, all students, demonstrated a small increase in mathematics self-concept over the course of the longitudinal study. We would have expected the age-related decline reported in prior research (e.g., Wilkins, 2004) to have occurred, but it did not. Thus, we suggest that the pedagogical practices associated with teachers having high expectations of all their students appeared to be positively associated with an amelioration of the expected decline.

Mathematics self-concept is not only shaped by messages received from influential others, for example, teachers (Dickhäuser and Stiensmeier-Pelster, 2003), but also social comparison with peers (Parker et al., 2014). Peer comparison acts to diminish levels of mathematics self-concept as age advances (Wilkins, 2004). Pedagogical elements of the intervention promoted positive messages from teachers (as influential others) and created classroom climates where social comparison with peers was discouraged. In support of this idea, McDonald et al. (2014) reported that teachers’ responses regarding the implementation of the current intervention acknowledged increased student self-belief and self-confidence. Teachers indicated that mixed ability grouping, promoting choice of learning activity, facilitation of a collaborative rather than competitive class climate, teacher positivity, and a buddy-system where students encouraged peer responsibility for the classroom climate fostered ownership of student learning and enabled the celebration of success for each child (McDonald et al., 2014). These changes to teacher beliefs and practices, taught during the intervention, may have created a learning environment that lessened the effect of social comparison as a source of age-related decline in mathematics self-concept.

Although the intervention appeared to be positively associated with student mathematics self-concept overall, neither the gender gap in mathematics self-concept, which favored boys, nor differences in the construct between ethnic groups, which positioned Māori in New Zealand at a particular disadvantage, were altered. Despite finding that mathematics self-concept rose slightly for students overall, girls’ mathematics self-concept was comparatively lower than boys’, and this gap persisted despite the intervention. The presence of a gender gap in mathematics self-concept supported previous New Zealand findings (e.g., Bonne, 2016) yet suggested that further changes to teacher beliefs and practices (specifically regarding gender) over and above those made during the current study were necessary. Importantly, in terms of gender equity, attention to mathematics achievement will not promote girls’ trajectories toward STEM pathways and careers without an accompanying self-concept that bolsters belief of success (Goldman and Penner, 2014).

Stereotypical notions of mathematics ability for girls have been reinforced by teachers with negative outcomes and are strongly related to girls’ mathematics self-concept (Ertl et al., 2017). Further, self-concept relies on the part of one’s perception of one’s self that is activated (Kessels and Hannover, 2008). Given the aforementioned research, it could be suggested that Kessels and Hannovers’ (2008) advocacy for reducing gender salience in class climates, should be considered. Thus, raising teacher awareness of gender salience in classrooms seems an important adjunct to challenging teacher beliefs and practices. Such a step may reduce the accessibility of gender-related self-descriptions and, thus, the impact of gender-related stereotypes, with the potential to impact mathematics self-concept (see Kessels and Hannover, 2008). Teachers (and parents) can help girls to build their self-beliefs and confidence in mathematics by realistic evaluation of girls’ actual abilities rather than recourse to their own beliefs about girls’ potential in the subject (OECD, 2015). Further, girls’ mathematics self-beliefs could be bolstered by feedback to combat cultural messages that privilege males in relation to mathematics (Correll, 2001).

The greatest gaps in mathematics self-concept have been found in countries with the most pronounced norms of gender-role rigidity and New Zealand has been identified among these (Hofstede, 2003). Further, New Zealand society has been described as gender-essentialist, that is, clearly demarking masculine and feminine attributes and roles as discreet from and opposite to each other in nature (Cushman, 2008). It seems unsurprising, therefore, that a gender gap in mathematics self-concept persisted in the current data. Training teachers to identify their own gender biases (see OECD, 2015) could further enhance interventions to raise teacher expectations. Such actions could ensure that not only enhanced student achievement but, critically (and with important implications for girls’ future capacity for STEM involvement), equitable self-concept in mathematics, were addressed.

Levels of mathematics self-concept for New Zealand European, Asian, and Pacific Island students rose slightly within the context of the intervention (after controlling for achievement differences). Māori students’ mathematics self-concept, however, continued to decline across the 3 years of the current study. We might have expected that Māori students, being from a collectivist cultural background (Harrington and Liu, 2002), would have been favorably affected by the promotion of a collaborative rather than competitive class climate, taught to teachers during the intervention. Rothstein-Fisch et al. (1999) found that within a collectivist context, one child’s mathematics success became success for the whole group, that the whole group celebrated individual success, and that these student behaviors were promoted in mixed-ability classrooms. Yet, although Asian students’ mathematics self-concept grew, that of Māori students diminished.

Previous research (Rubie-Davies et al., 2006) has found that teachers have expected less of Māori students. Importantly, teachers have placed the onus for such expectations on deficits attributed to the Māori students themselves and their cultural background (Bishop et al., 2010). Bishop et al. (2010) described *whanaungatanga* (the warmth and closeness associated with the extended family) reflected in relationships within the classroom, and especially those between the student and teacher, as key to Māori students’ success. Notably, Māori students wished to be acknowledged positively by their teachers as Māori (Bishop et al., 2010). The centrality of successful teacher-student relationships for positive student outcomes (Hattie, 2009) was a feature of the current intervention, and here again we would have expected this to have been associated with positive outcomes for all students. In line with the suggestions of Rubie-Davies and Peterson (2016), we suggest that specific attention seems needed to further raise teachers’ awareness of culturally responsive pedagogy uniquely tailored to the needs of Māori students.

### Limitations and Directions for Future Research

The current study was not conclusively able to deduce that it was the intervention and not confounding elements that were related to the amelioration of the decline in mathematics self-concept over time. Future research could test the effectiveness of pedagogical practices deemed likely to have reduced, for example, the social comparison often held responsible for the age-related decline in the construct. Further, the current sample suffered from significant attrition and comprised only participants from one urban center, which may reduce generalizability of the findings. Although attrition would be hard to address given the schooling structure of New Zealand, other urban locations and rural settings could be investigated in the future. As well, the current intervention did not set out to address mathematics self-concept as such, but the findings suggest that further attention to nuanced teacher beliefs encompassing gender and ethnicity, could be valuable in future iterations. Additional training to help teachers recognize and confront biases they may hold about different groups of students (e.g., in terms of gender, and ethnicity) may aid teacher effectiveness and further enhance student potential (OECD, 2015).

### Conclusion

The current paper aimed to explore whether, within the context of an intervention to raise and sustain teacher expectations, the amelioration of an age-related decline and a gender gap in mathematics self-concept would be observed and equitable levels of mathematics self-concept across ethnic groups would be found. The finding that mathematics self-concept over time improved (where a decline had been expected) suggests that comprehensively addressing social comparison issues within class climates may be adaptive in fostering positive levels of the construct. Nevertheless, findings that a gender gap favoring boys persisted and a notable decline occurred for Māori students suggest the need for specific interventions to address gender-related issues and to nurture Māori students’ perceptions of themselves as mathematicians. Notably, the findings point to the importance of considering context in the study of mathematics self-concept in order to successfully implement future interventions.

## Ethics Statement

Permission to proceed with the current research was granted by the University of Auckland Human Participants Ethics Committee. Consent to invite teacher and student participation was gained from all participating school Principals. All participating teachers fromalized their consent, and their students (all being under the age of 16) could assent to participation once their parents had given consent that they may be invited to take part in the study. Anonymity and confidentiality regarding all participants and participating schools, were rigorously preserved.

## Author Contributions

All authors listed have made a substantial, direct and intellectual contribution to the work, and approved it for publication.

## Conflict of Interest

The authors declare that the research was conducted in the absence of any commercial or financial relationships that could be construed as a potential conflict of interest.

## Footnotes

1 http://e-asttle.tki.org.nz/
